# Review on the Evaluation and Development of Artificial Intelligence for COVID-19 Containment

**DOI:** 10.3390/s23010527

**Published:** 2023-01-03

**Authors:** Md. Mahadi Hasan, Muhammad Usama Islam, Muhammad Jafar Sadeq, Wai-Keung Fung, Jasim Uddin

**Affiliations:** 1 Department of Computer Science and Engineering, Asian University of Bangladesh, Ashulia 1349, Bangladesh; 2 School of Computing and Informatics, University of Louisiana at Lafayette, Lafayette, LA 70504, USA; 3 Department of Applied Computing and Engineering, Cardiff School of Technologies, Cardiff Metropolitan University, Cardiff CF5 2YB, UK

**Keywords:** COVID-19, artificial intelligence, machine learning, deep learning, few-shot learning

## Abstract

Artificial intelligence has significantly enhanced the research paradigm and spectrum with a substantiated promise of continuous applicability in the real world domain. Artificial intelligence, the driving force of the current technological revolution, has been used in many frontiers, including education, security, gaming, finance, robotics, autonomous systems, entertainment, and most importantly the healthcare sector. With the rise of the COVID-19 pandemic, several prediction and detection methods using artificial intelligence have been employed to understand, forecast, handle, and curtail the ensuing threats. In this study, the most recent related publications, methodologies and medical reports were investigated with the purpose of studying artificial intelligence’s role in the pandemic. This study presents a comprehensive review of artificial intelligence with specific attention to machine learning, deep learning, image processing, object detection, image segmentation, and few-shot learning studies that were utilized in several tasks related to COVID-19. In particular, genetic analysis, medical image analysis, clinical data analysis, sound analysis, biomedical data classification, socio-demographic data analysis, anomaly detection, health monitoring, personal protective equipment (PPE) observation, social control, and COVID-19 patients’ mortality risk approaches were used in this study to forecast the threatening factors of COVID-19. This study demonstrates that artificial-intelligence-based algorithms integrated into Internet of Things wearable devices were quite effective and efficient in COVID-19 detection and forecasting insights which were actionable through wide usage. The results produced by the study prove that artificial intelligence is a promising arena of research that can be applied for disease prognosis, disease forecasting, drug discovery, and to the development of the healthcare sector on a global scale. We prove that artificial intelligence indeed played a significantly important role in helping to fight against COVID-19, and the insightful knowledge provided here could be extremely beneficial for practitioners and research experts in the healthcare domain to implement the artificial-intelligence-based systems in curbing the next pandemic or healthcare disaster.

## 1. Introduction

The severe acute respiratory syndrome coronavirus 2 (SARS-CoV-2) is an extremely contagious disease that was detected for the very first time on 31 December 2019, Wuhan, China [1]. It subsequently spread internationally, causing more than 547 million confirmed infections and 6.33 million deaths, making it one of the worst diseases in human history.

Pre-COVID-19, there were three major epidemics in the twenty-first century, starting with severe acute respiratory syndrome coronavirus (SARS-CoV) in 2002–2003. The second one was swine flu in 2009–2010, and the third one was Middle East respiratory syndrome coronavirus (MERS-CoV), which started in 2015 [2]. With various warning shots for the research community [3,4] relevant to coronavirus and its potential to cause human harm, it was quite inconceivable that another pandemic was imminent. COVID-19 has proven to be a challenging sickness that could present in a variety of forms and degrees of severity, ranging from mild to severe (organ failure and death). Multi-organ failure and death are unusual from a mild, self-limiting respiratory disease, but it can present as a severe, progressive pneumonia. With the progression of the pandemic and the increasing number of verified cases and people suffering from severe respiratory failure and cardiovascular issues, there are strong grounds to be very worried about the effects of this viral infection [5]. A considerable amount of attention has been placed on identifying acceptable strategies for addressing COVID-19-related challenges. Artificial intelligence has recently attracted much research effort towards solving the complex issues in a number of fields, including engineering [6,7,8], medicine [9,10], economics [4], and psychology [11].

From the molecular level to the most up-to-date data-driven decision-making models, research literature and digital technologies have had significant impacts [12,13]. They are based on the technological advancement of artificial intelligence, which is inspired by the human brain. Artificial intelligence is the use of algorithms, models, and computer techniques to realize human intelligence [14]. Significant advancements in processing power, virtual (algorithm) dimensions, numerical optimization, and memory have enabled the creation and implementation of cutting-edge AI solutions to combat COVID-19 throughout the past decade. Due to their ability to swiftly adapt to ever-changing inputs, artificial intelligence systems are useful in situations of rapid change.

Machine learning is the subset of artificial intelligence that has the ability to learn from and make predictions based on data. Deep learning is a subset of machine learning that uses data, weights, hyperparameters, and complex structures of algorithms modeled on the human brain. Machine learning and deep learning have reached important milestones in processing, complicated decision making, information analysis, and extremely organized self-learning.

Several research themes are in spotlight due to COVID-19. Alballa et al. [15] exclusively examined machine learning strategies for diagnosis, mortality, and severity risk prediction. Napolitano et al. [12] provided an overview of COVID-19 applications, including molecular virology, molecular pharmacology and biomarkers, epidemiology, clinical medicine, clinical imaging, and AI-based healthcare. The authors have not clarified the limitations they discovered in the previously mentioned areas. El-Rashidy et al. [16] explored applications of artificial intelligence for COVID-19. This research centered on COVID-19 diagnosis, spread, features, treatment, and vaccine development; it included a few supporting applications. Alyasseri et al. [17] reviewed deep learning (DL) and machine learning (ML) techniques for only COVID-19 diagnosis. Kwekha-Rashid et al. [18] reported their study of coronavirus illness cases using just ML (supervised and unsupervised) techniques. Current restrictions and prospective scopes have not been defined by the authors. Bhattacharya et al. [19] and Roberts et al. [20] highlighted medical image processing applications for COVID-19 that use deep learning and machine learning methods. We investigated supervised learning techniques for COVID-19 forecasting, clinical-feature-based COVID-19 prediction, medical-image-based COVID-19 detection, the immunological landscape of COVID-19 analysis, and COVID-19 patients’ mortality-risk prediction, among others. For unsupervised learning, we show exploratory medical image grouping, risk analysis, anomaly detection, differentiation, patient severity detection, patient screening, and discovery of disease-related genes. Object detection approaches we found are for COVID-19 detection and screening, infection risk assessment, abnormality detection, body temperature measurement, personal protective equipment detection, and social distance monitoring.

The transfer learning-based COVID-19 study we investigated included automatic analysis of medical pictures, COVID-19 classification, identification of lung-disease severity, and an automated COVID-19 screening model. Medical images based on COVID-19 infected area segmentation, lung and infection region segmentation, and infected tissue segmentation are key applications in the image segmentation sector. Detection of COVID-19 and medical image analysis are the primary applications in shot learning arena.

Our research carefully examines AI applications’ current status, identifies technical issues and limitations, and provides instructions to overcome existing challenges. Moreover, this comprehensive investigation will assist the research community in the upcoming development of artificial intelligence technology for any pandemic.

**Figure 1:**
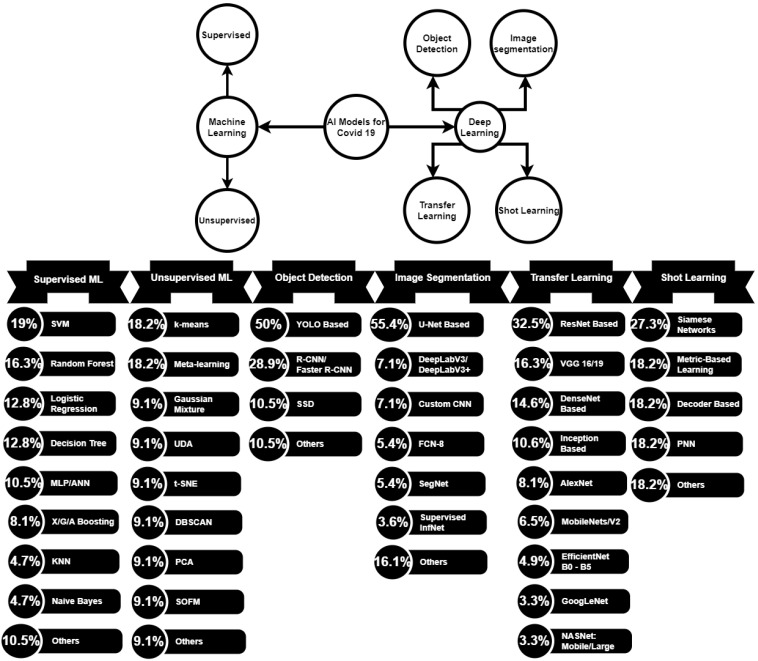
The infographic overview of AI approaches for COVID-19. The percentage indicates the use of AI models in diverse areas of COVID-19.

**Figure 2:**
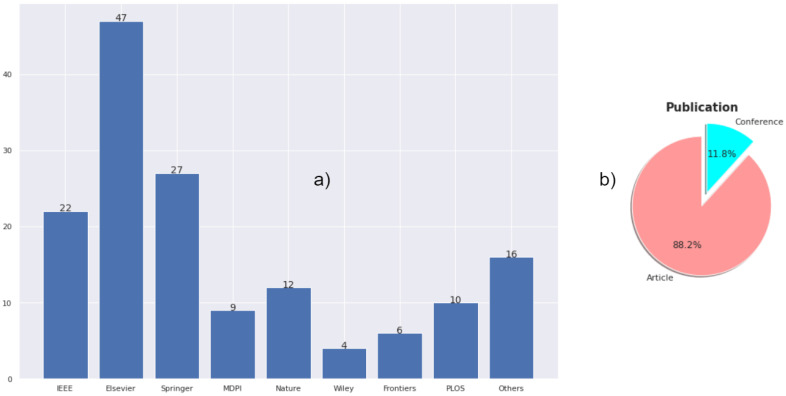
Overview of papers selected from different databases. (**a**) The bar plot illustrates the numbers of papers selected from Nature, IEEE, Springer, Elsevier, MDPI, PLOS, Wily, Frontiers, and others. (**b**) The pie chart shows the distribution of selected articles and conferences.

**Figure 3:**
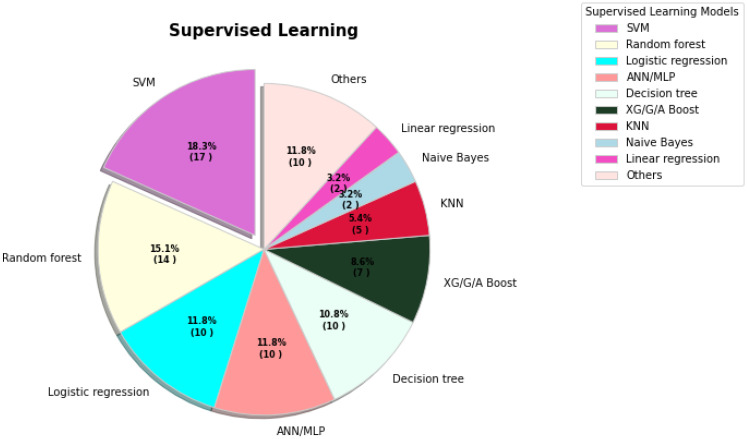
The percentages of various supervised learning models being used for COVID-19 tasks.

**Figure 4:**
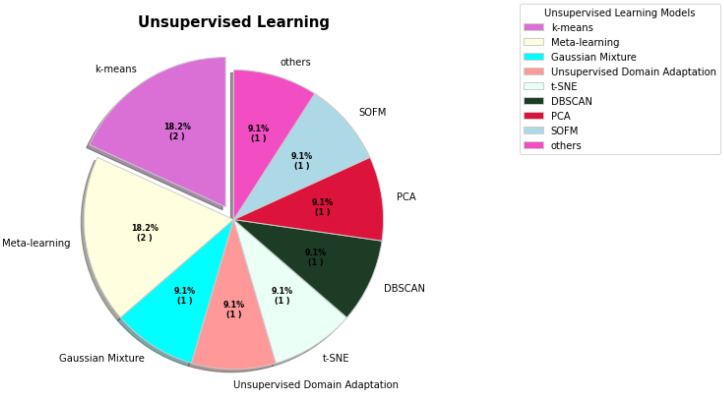
The percentages of various unsupervised learning models used for COVID-19.

**Figure 5:**
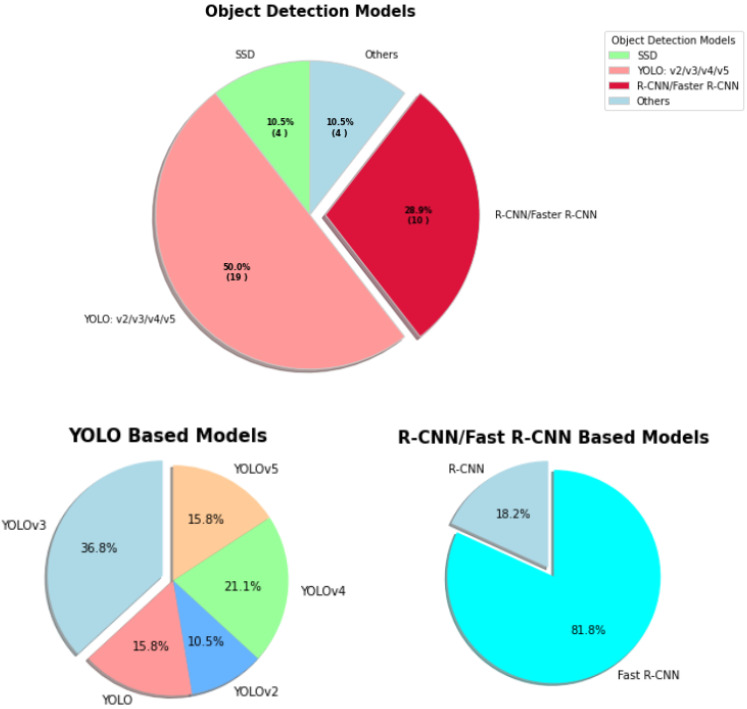
The rate of percentages are used in various object detection models for COVID-19. The most used models are shown in a sub-chart for proper visualization.

**Figure 6:**
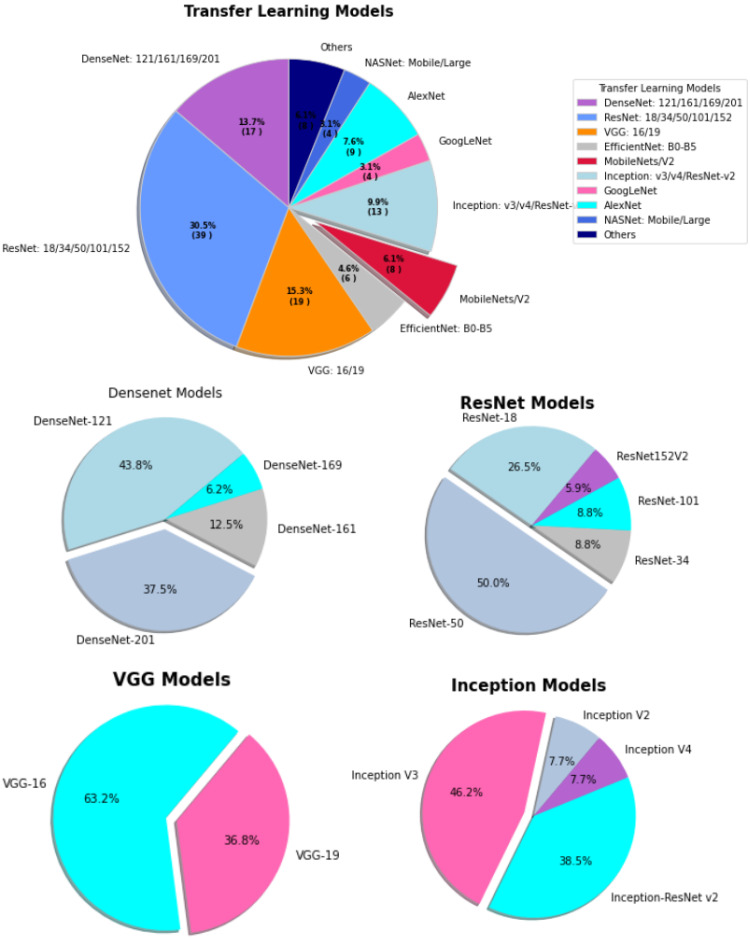
The percentages of various transfer learning models used for COVID-19.

**Figure 7:**
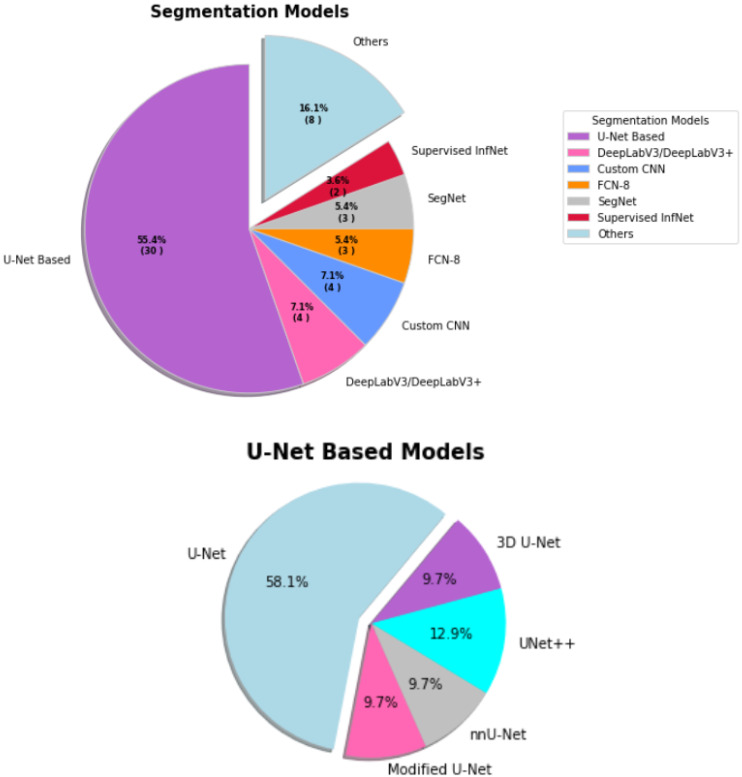
The percentages of segmentation models applied for COVID-19.

**Figure 8:**
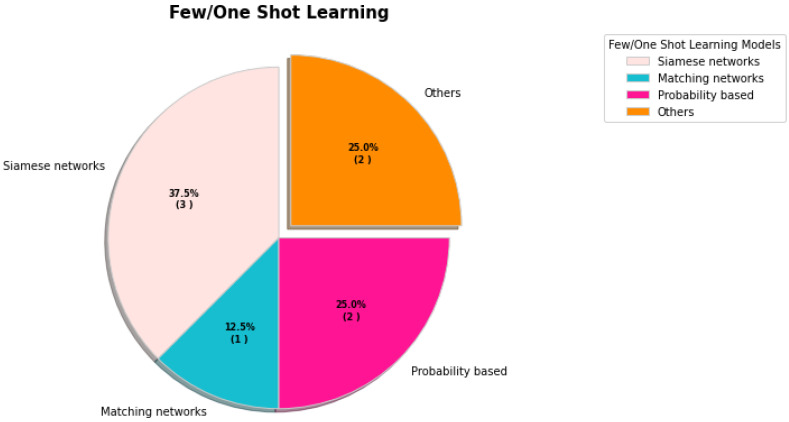
The percentage of shot learning models used for COVID-19.

The rest of this paper consists of the following sections. Section 2 presents the methodology of the research. Section 3 presents the supervised and unsupervised learning applications for COVID-19. Section 4 presents object detection, transfer learning, image segmentation, and shot learning applications for COVID-19. Section 5 encompasses discussion and future work. Section 6 concludes the paper. Figure 1 shows overview of AI approaches for COVID-19. Figure 2, Figure 3, Figure 4, Figure 5, Figure 6, Figure 7 and Figure 8 are infographics overviews. Table 1, Table 2, Table 3 and Table 4 are tabulated summaries. Table 5 includes a meta-analysis for specific objectives. Table 6 includes current challenges and future research directions.

## 2. Methodology

A multi-step scanning strategy [21] was applied for this systematic review. We searched relevant published literature from 2020 to 2022. Search strings consisting of twelve keywords—namely, ‘standards’, ‘report criteria’, ‘COVID-19’, ‘artificial intelligence’, ‘machine learning’, ‘deep learning’, ‘supervised learning’, ‘unsupervised learning’, ‘transfer learning’, ‘object detection’, ‘image segmentation’, and ‘few-shot learning’—in several combinations were used. We only searched literature written in English. Initially, we selected 302 papers that matched our research criteria and after removing duplicates retained 239. Retained papers were filtered based on titles, abstracts, contexts, and the scope of our domain. Only 150 papers were deemed relevant to our review. We scanned the full-text and removed ineligible papers. In addition, we included 128 papers for review. Figure 2 shows the overview of databases that were used in this review.

### Eligibility Criteria

The selection and filtering process is summarized as follows:

- The research must have discussed AI-based devices or architectures, or systems for forecasting or detecting or monitoring or managing health care conditions for COVID patients.
- A clear methodology must have been coined in terms of devices used or architectures discussed in the studies.
- Unique, relevant, important, significant, and informative works were included.
- Relevant papers with historical insights were included in the discussion to find the current state of research, past historical evidence, and future policy implications for future endemics.
- Duplicate research works were excluded.
- Research work without IRB approval where a human subject was directly involved in the study were discarded.
- Media reports, university reports, and reports with ambiguity were excluded due to lack of clear methodology.
- Journal ranking (Q1≥Q2≥Q3≥Q4≥no−Q), impact factor, JCR, and conference ranking by ERA and Quails (A≥B≥C≥Unranked) were prioritized. Papers belonging to predatory journals were usually discarded to the best of our knowledge.

## 3. Machine Learning

### 3.1. Supervised Learning

The supervised learning technique examines the training data and develops the hypothesized function to map new instances. The supervised learning-based COVID-19 studies concentrate on several fields, such as COVID-19 forecasting, clinical-feature-based COVID-19 prediction, medical-image-based COVID-19 detection, the immunological landscape of COVID-19 analysis, and mortality-risk prediction of COVID-19 patients, among others.

Cabitza et al. [22] developed blood tests that are inexpensive and deliver results quickly for identifying COVID-19. The authors applied hematochemical results from 1624 patients hospitalized at San Raphael Hospital (52% were COVID-19 positive). For classification purposes, they employed a variety of machine learning methods, including logistic regression, naive bayes, k-nearest neighbor (KNN), random forest, and support vector machine (SVM). KNN had the highest accuracy. Satu et al. [23] created a COVID-19 short-term forecasting system. The authors researched instances of COVID-19 infection in Bangladesh. The dataset was taken from the Johns Hopkins University Center for Systems Science and Engineering’s GitHub repository. Several machine learning methods, such as support vector regression (SVR), polynomial regression, linear regression, polynomial multilayer perceptron (PMLP), multilayer perceptron (MLP), and prophet, were employed to anticipate the numbers of infected and fatal cases. Prophet had the lowest error rate for predicting the following seven days of infection and mortality cases.

Arpaci et al. [24] developed a clinical-characteristics-based method for predicting COVID-19 infection. The dataset included 114 cases gathered from the hospital in Taizhou, Zhejiang Province, China. Positive and negative classes are present in the dataset. On the basis of fourteen clinical characteristics, they utilized six classic classifiers, including a Bayes classifier, meta-classifier, rule learner, decision tree, lazy classifier, and logistic regression. The meta-classifier was accurate 84.21% percent of the time. The mortality prediction approach for COVID-19 patients was examined by Chowdhury et al. [25]. The authors utilized a dataset of 375 COVID-19-positive patients admitted to Tongji Hospital (China) between 10 January and 18 February 2020. In addition, they studied the demographics, clinical features, and patient outcomes of COVID-19-positive patients. They utilized the XGBoost algorithm with several trees to forecast patient risk. Patterson et al. [26] developed cytokines of the immunological landscape of COVID-19 utilizing algorithms for machine learning. The synthetic oversampling approach waws applied to address the imbalance issue. The model consists of three components: the severe disease binary classifier, the multi-class predictor, and the post-acute sequelae of COVID-19 (PASC) binary classifier. The authors classified data using the random forest algorithm. The accuracy of the binary classifier for severe illness was 95%, the accuracy of the multi-class predictor was 80%, and the accuracy of the PASC binary classifier was 96%.

To enhance patient care, Karthikeyan et al. [27] presented a COVID-19 mortality-risk prediction approach. The dataset contained 2779 computerized records of COVID-19-infected or suspected-to-be-infected patients from Tongji Hospital in Wuhan, China. The prediction models utilized a combination of lactate dehydrogenase (LDH), neutrophils, lymphocytes, high-sensitivity C-reactive protein (hs-CRP), and age extracted from blood test data. For risk prediction purposes, the researcher used neural networks, XGBoost, SVM, logistic regression, decision trees, and random forests. Compared to XGBoost, SVM, logistic regression, decision trees, and random forests, the neural network obtained higher accuracy. Marcos et al. [28], discovered early identifiers for patients who would die or require mechanical breathing during hospitalization. The dataset consisted of 1260 verified COVID-19 patients from Spain’s University Hospital of Salamanca and Hospital Clinic of Barcelona. Their decision-making approach is based on clinical and laboratory characteristics. The authors utilized three different classifiers: logistic regression, random forest, and XGBoost. The three models attained area under the curve (AUC) ratings of 83 percent, 81 percent, and 82 percent, respectively. The COVID-19 patient mortality prediction system was developed by Mahdavi et al. [29]. Between 20 February 2020 and 4 May 2020, the authors analyzed the electronic medical data of 628 patients at Masih Daneshvari Hospital. The dataset is divided into three sections: clinical, demographic, and laboratory. Three SVM models were utilized by the authors. The steps were: First, providing clinical and demographic information. The second step is to enter clinical, demographic, and laboratory information. Third, just enter laboratory data. Three linked models attained the highest degree of precision.

Li et al. [30] established a COVID-19 patient mortality prediction system. In Wuhan, China, the authors collected clinical data from COVID-19 patients. They constructed three classification-based models. Model one was a decision tree and gradient boosting classifier combination. Model two was a logistic regression classifier. Model three was a logistic regression classifier with three or five features. The best accuracy was attained by the decision tree with gradient boosting. The serum-glucose-based COVID-19 prediction method was introduced by Podder et al. [31]. There are 5644 rows and 111 columns in the dataset obtained from Hospital Israelita Albert Einstein in Sao Paulo, Brazil. The dataset contains significant shortcomings, such as an imbalance between classes and missing values. To balance the dataset, the undersampling approach was utilized. For classification objectives, XGBoost, random forest, decision tree, and logistic regression were utilized. The XGBoost algorithm outperformed the competition.

Chandra et al. [32] explored a categorization approach based on chest X-ray images. COVID-Chestxray set, Montgomery set, and NIH ChestX-ray14 set are three public repositories with which datasets were compiled. The authors distinguished between nCOVID and pneumonia, and normal and aberrant. They utilized the artificial neural network, support vector machine (kernel: RBF, poly, linear), decision tree, k-nearest neighbor, and naive bayes for classification purposes. Additionally, they utilized the majority voting algorithm. Among the previously described models, the majority vote algorithm performed the best. The short-term cumulative COVID-19 case forecasting model was introduced by Balli et al. [33]. The dataset comprises the weekly confirmed case and cumulative confirmed case data compiled by the World Health Organization. They utilized linear regression, MLP, random forest, and SVM to forecast the pandemic trend. SVM has the best trend among the listed algorithms. Li et al. [34] found unique risk variables for COVID-19 patients. In order to train the model, the LASSO (least absolute shrinkage and selection operator) technique was used. The authors have added blood types such as B and AB as protective variables, and A as a risk factor. In addition to age, gender, temperature, humidity, health expenditure, social distance, smoking, urbanization level, and race, they found a number of other characteristics.

Kang et al. [35] established a prediction model based on clinical data for COVID-19 patients with severe symptoms. China’s Tongji Medical College-affiliated Union Hospital’s Tumor Center created the dataset. This dataset includes 151 instances between 26 January and 20 March 2020. They created a four-layer ANN model with six nodes in the input layer, thirteen and thirteen nodes in the two hidden levels, and one node in the output layer. The ANN model attained an average of 96.9 percent accuracy.

The clinical prognostic evaluation of COVID-19 patients was proposed by Kocadagli et al. [36]. The COVID-19 patient dataset was retrieved from Koc University Hospital in Istanbul, Turkey. The data collection included symptoms, demographic features, blood test results, and illness histories from individuals of all ages and genders. For purposes of classification, ANNs, SVMs, and AdaBoost (weak learner: decision trees) were implemented. The best accuracy was reached by ANNs. Udhaya Sankar et al. [37] investigated mobile voice analytic applications for COVID-19 detection. The authors did not specify the methods used or the performances of those algorithms. Gokcen et al. [38] created artificial neural networks for detecting COVID-19 using cough data. Experiments have utilized available speech data from the Massachusetts Institute of Technology (MIT). Using a filter, cough sounds were cleaned, and the mel-frequency cepstral coefficient was applied to extract characteristics. The model is comprised of four layers, with 256, 128, 64, and 1 neurons in each. The accuracy of the model was 79 percent.

Nalini et al. [39] have explored sentiment analysis of COVID-19 from Twitter. The dataset consists of 3090 tweets in four classes: fear, sad, anger, and joy. They created four models: bidirectional encoder representations from transformers (BERT), logistic regression (LR), support vector machines (SVM), and long-short-term memory (LSTM). Models produced accuracies of 89%, 75%, 74.75%, and 65% respectively.

**Table 1:** The systematic overview of problems and solutions addressed through the supervised learning methods for COVID-19.

Ref.	Problem Definition	ML Models	Sample	Performance
[40]	Prediction of COVID-19 infection	Logistic regresseaturion, Decision tree, SVM, naive Bayes, ANN	RT-PCR test 263,007 records, 41 features	Accuracy: 94.41%, 94.99%, 92.4%, 94.36%, 89.2%
[41]	The number of the positive cases prediction method	Nonlinear regression, decision tree, random forest	Six features (deaths, recovered, confirmed, amount of testing, lockdown, lockdown features)	MAPE: 0.24%, 0.18%, 0.02%.
[42]	Prediction model for mortality in COVID-19 infection	SVM	398 patients (43 expired and 355 non-expired)	Sensitivity: 91%, specificity: 91%
[43]	COVID-19 computed tomography scan dataset for ML	–	169 patients positive, 76 normal patients, and 60 patients with CAP	–
[44]	Risk factors analysis of COVID-19 patients and ARDS or no-ARDS prediction method	Decision tree, logistic regression, random forest, SVM, DNN	659 COVID-19 patients and clinical features	Accuracy: 97%, 93%, 92%, 83%, 90%
[45]	Patient intensive care and mechanical ventilation prediction method	Random Forest	Socio-demographic, clinical data 212 patients (123 males, 89 females)	AUC: 80%, AUC: 82%
[46]	Prediction of COVID-19 diagnosis based on symptoms	Gradient-boosting	Test records of 5183 individual (cough, fever, sore throat, shortness of breath, etc)	Sensitivity: 87.30%, specificity: 71.98%
[20]	Early risk identification of (SARS-CoV-2) patients	Logistic regression, decision tree, random forest, KNN, SVM, AdaBoost, MLP	Total 198 patients (135 non-severe, 63 severe COVID-19)	SVM: median 96%. Other model performance result unclear in the paper
[47]	Chest X-ray images based COVID-19 infection detection	KNN, decision tree, random forest, L-SVC, SVC	371 positive, 1341 normal chest X-ray images	Precision: 98.96%, 94.89%, 97.58%, 99.3%, 99.66%
[48]	SARS-CoV-2 pre-miRNAs detection	KNN, RUNN-COV, logistic regression, random forest, SVM	positive 569 and negative 999,888 pre-miRNA samples	F1 score: 89.86%, 98.26%, 89.47%, 91.55%, 89.83%

An LSTM network-based COVID-19 cases and deaths forecast system was designed by Yogesh [49]. The dataset was collected from the source (https://ourworldindata.org/, accessed on 20 December 2022). The dataset contains information about cases and deaths in Italy, France, Brazil, India, Germany, United States, and Nepal. According to the day, there were two steps: singlestep and multistep. Multistep models had higher error values than singlestep models. Social media-based COVID-19 sentiment analysis was done by Mohamed et al. [50]. A bidirectional long short-term memory (Bi-LSTM) model was employed for classification purposes. The authors used three datasets obtained from Twitter and Reddit platforms. The dataset collection duration was 2017 to 2020. The two Twitter datasets contain 505,243 tweets and several labels (negative, neutral, positive, fun, surprise, etc). On the other hand, the Reddit one contains 563,079 COVID-19-related comments. It has five labels (very positive, positive, neutral, negative, and very negative). The proposed Bi-LSTM model has several layers, such as embedding, bidirectional, dropout, and dense layers. The model produces highly fluctuating F1 scores.

Pahar et al. [51] categorized the COVID-19 cough using machine learning. The authors acquired the dataset via an online site. The normalization method was employed for data preparation. Features were extracted using mel-frequency cepstral coefficients, log frame energies, zero-crossing rate, and kurtosis. The support vector machine, logistic regression, k-nearest neighbor, multilayer perceptron, convolutional neural network, long short-term memory, and residual-based neural network architecture (Resnet50) were utilized for classification applications. The conventional machine learning techniques (support vector machine, logistic regression, k-nearest neighbor, and multilayer perceptron) are extremely inefficient.

In supervised learning, Figure 3 shows the SVM contributed highest segment. In addition, random forest, logistic regression, ANN/MLP, and decision tree contributed 15.1%, 11.8%, 11.8%, and 10.8% respectively. Table 1 presents problems and solutions through the supervised learning methods for COVID-19.

### 3.2. Unsupervised Learning

Models of unsupervised learning find hidden objects, patterns, and groupings without data labels or human interaction. Unsupervised models provide a solution for exploratory medical picture grouping, risk analysis, anomaly detection, differentiation, patient severity detection, patient screening, and discovery of disease-related genes.

Boussen et al. [52] created a clustering-based COVID-19 patient severity and intubation monitoring system. Green, orange, and red are the classifications for the patient monitoring and decision-making sections, respectively. Each group has distinct characteristics, such as a lesser risk of intubation, a high degree of hypoxia, and the prompt consideration of intubation for a patient in the red category. Gaussian mixture represents the clustering model. The model achieved 87.8 percent accuracy. Zhao et al. [53] built an unsupervised model to identify anomalous changes in PM2.5 in Chinese cities between 2017 and 2020. The dataset is comprised of 9,721,023 samples. Encoder, decoder, and anomaly assessment make up the method’s three blocks. This technology can help monitor air quality in response to abrupt changes. Lai et al. [54] evaluated publicly accessible and pertinent COVID-19 data sources, addressed the difficulty of data heterogeneity by clustering, and categorized counties based on underlying variations. For clustering purposes, the k-means clustering method was implemented.

Chen et al. [55] investigated the segmentation network for the COVID-19 computed tomography (CT) infection. Data were compiled from the Italian Society of Medicine, International Radiology, and prior research. Their dataset consists of 10,200 2D CT scans synthesized in a laboratory. The authors utilized the unsupervised domain adaptation learning architecture. The proposed network is divided into multiple modules, including a feature extractor, pixel-wise classifier, and domain adaption module. Convolutional and max-pooling layers were employed to extract features. The network’s dice performance was 86.54 percent, its sensitivity was 85.54 percent, and its specificity was 99.80 percent. Kurniawan et al. [56] introduced a clustering and correlation matrix-based COVID-19 risk analysis technique for pandemic nations. The authors retrieved information from Worldometers. For clustering applications, the k-means method has been used. Five experiments were conducted based on varying numbers of clusters. Five was the optimal number of clusters.

Zheng et al. [57] investigated unsupervised meta-learning for distinguishing COVID-19 and pneumonia patients. They presented a dataset consisting of 2696 images of COVID-19 pneumonia; and 10,155 images of SARS, MERS, influenza, and bacterial pneumonia. The data augmentation approach was employed to produce images. This framework was comprised of two modules: one based on network-based learning and the other on relational models. Utilizing the DenseNet-121 architecture, network-based learning characteristics were extracted. The relation model is represented by an 8-pooling layer network architecture. This model performs better than supervised models such DenseNet-121, DenseNet-161, ResNet-34, and VGG-19. Oniani et al. [58] investigated relationships between several biological entities and COVID-19. Experiments were conducted using the CORD-19-on-FHIR dataset. T-distributed stochastic neighbor (t-SNE) and the density-based clustering method (DBSCAN) were used for clustering purposes.

Ewen et al. [59] suggested online unsupervised learning approach for COVID-19-CT-scan image classification. The components of online unsupervised learning include online machine learning and unsupervised learning. In the experiment, the COVID-19-CT-scan-images dataset from the signal processing grand challenge (SPGC) was utilized. The dataset includes three categories: healthy, COVID-19, and CAP. The technique of horizontally flipped data augmentation was used to increase the number of image copies. The baseline adopted the DenseNet169 architecture. The accuracy of the proposed model was 86.7%.

Miao et al. [60] created an unsupervised meta-learning model for the screening of COVID-19 patients. The author gathered three datasets from publicly accessible sources, including BIMCV-COVID19+, Kaggle-pneumonia, and CC-CXRI. The unsupervised meta-learning model was composed of both the DL model and gradient-based optimization. Convolution, max-pooling, and batch normalization are a few of the DL model’s layers. This model is superior to the LeNet, Alexnet, visual geometry group (VGG), CovXNet CNN-RNN, and EMCNet models.

Fujisawa et al. [61] examined the COVID-19 disease-related gene identification approach using unsupervised main-component-analysis-based feature extraction (PCAUFE). PCAUFE was applied to the RNA expression patterns of 18 healthy individuals and 16 patients with COVID-19. The expression of RNA yielded the identification of 123 genes. The authors classified COVID-19 patients and non-patients based on 123 genes identified by PCAUFE using logistic regression (LR), support vector machine (SVM), and random forest (RF) models. Three models attained areas under the curve (AUC) in excess of 90%.

King et al. [62] investigated the grouping of COVID-19 chest X-ray images using self-organizing feature maps (SOFM). The authors obtained the dataset from a freely accessible source. This dataset consists of two classes: infected and non-infected. They compared and measured the distributions of pixels in the non-infected cluster and the infected cluster using the overlapping coefficient.

Xu et al. [63] developed an unsupervised technique for lung segmentation and pulmonary opacity identification using CT scan images. The datasets were obtained from Osaka University, Zhejiang University, Hangzhou Second People’s Hospital, Jingmen First People’s Hospital, Taizhou Hospital, and Sir Run Shaw Affiliated Hospital of Zhejiang University School of Medicine, among others. The image augmentations approach was utilized to produce training images. The lung segmentation model had U-shaped convolutional neural network (U-Net) architecture and many pre-trained encoders, including VGG19, MobileNetV2, and ResNet50. For opacity detection, an auto-encoder based on generative adversarial networks (GAN) was utilized. Classification techniques utilizing support vector machine, random forest, adaptive boosting, and XGBoost were compared. Overall, the accuracy of this method was 95.5%.

In unsupervised learning, Figure 4 indicates k-means and meta-learning reached 18.2%. However, gaussian mixture, unsupervised domain adaptation, t-SNE, DBSCAN, PCA, SOFM, and other models contributed 9.1%.

## 4. Deep Learning

### 4.1. Object Detection

Every object has distinguishing qualities and unique characteristics. The object detection methods are comprised of mathematical models and millions of parameters that are used to learn characteristics from objects and discover new instances. The object detection models have demonstrated possible applications in the COVID-19 domain, such as COVID-19 detection and screening, infection risk assessment, abnormality detection, body temperature measurement, personal protective equipment detection, and social distance monitoring.

Huang et al. [64] created a YOLO v4-based object detection method for multiplexed circular-flow immunoassay test strips that can rapidly measure and distinguish antibodies that bind the membrane glycoprotein of severe acute respiratory syndrome coronavirus 2. The lateral flow immunoassays are comprised of many pads, including sample, conjugate, absorbent pads, and nitrocellulose membrane. To evaluate the model, the authors did not provide any assessment measures. Xu et al. [65] investigated a location-attention technique-based screening method to differentiate COVID-19 from influenza-A viral pneumonia (IAVP) and healthy patients using lung CT scans. The collection comprises 618 CT images: 219 with COVID-19, 224 with IAVP, and 175 from healthy individuals. Utilizing random clipping, up–down flipping, left—right flipping, and mirroring, photos were enhanced. The location-attention technique was introduced to the ResNet-18 backbone by the authors. The noisy OR Bayesian function has been utilized to assess infection kind and overall confidence score. The total accuracy of the method was 86.7 percent.

Bhuiyan et al. [66] presented detection methods for face masks. Web-sourced data collection included 300 mask and 300 no-mask entries. To recognize face masks, the you only look once version 3 (YOLOv3) method was implemented. The LabelIMG software tool was used for image annotation purposes. The model achieved a classification and detection accuracy of 96%. Face recognition, mask detection, and social distancing monitoring were adopted by Vergin et al. [67] to decrease the propagation of the virus and human participation. The dataset comprises 200 face picture samples, 700 individuals wearing masks, and 700 individuals without masks. The authors further utilized the Microsoft Common Objects in Context (MS-COCO) and Visual Object Classes Challenge 2007 and 2012 (VOC0712) datasets. Utilizing a binary patterns histograms technique to recognize the face, MobileNetV2 transfers knowledge architecture to detect masks and a single shot detector to monitor social distance. The article lacks performance reports. Sandeep et al. [68] established a mask-detection approach through AlexNet and CNN. However, the authors used two datasets, the Real-World Masked Face Dataset (RMFD) and CelebA. The CNN consists of fifteen layers, including convolutional, max-pooling, flatten, dropout, and dense. This paper did not have a confusion matrix; even though their accuracy and precision were extraordinary, recall was dramatically worse. Hybrid deep transfer learning and machine learning-based face mask detection model were developed by Mohamed et al. [69]. The datasets were collected from three different sources: RMFD, the Simulated Masked Face Dataset (SMFD), and Labeled Faces in the Wild (LFW). The model has two blocks: a feature extraction block and a decision-making block. Resnet50 was employed for extracting features and the rest of them for decision-making (decision trees, SVM). The model produced remarkable accuracy. Similar studies were done by Jonathan et al. [70], who created facial recognition systems individuals with and without face masks. The dataset consists of 13,359 images: 7067 with a face mask and 6292 without a face mask. MobileNetV2 was used for classification and FaceNet for face recognition purposes. The model achieved an overall accuracy of 99.52%.

Hou et al. [71] described a method for detecting social separation using the video frame. The YOLOv3 model was utilized to identify bounding box coordinates, object confidence, and class label probabilities. There are no assessment metrics presented. Al-Antari et al. [72] investigated the object detection technique for chest X-radiation (X-ray) images. COVID-19, masses, effusion, pneumonia, atelectasis, infiltration, pneumothorax, cardiomegaly, and nodules are the nine distinct items included in the dataset. Utilization of you only look once (YOLO) architecture for training purposes. The model obtained 90.67 percent detection accuracy and 97.40 percent classification accuracy. Al-Antari et al. [73] performed work that was similar. The collection is comprised of 326 chest X-ray images from two distinct sources: Qatar University and the public dataset. They have utilized the YOLO object detector as well. This computer-aided design (CAD) framework achieved detection and classification accuracy of 96.31 percent and 97.40 percent, respectively.

Rezaei et al. [74] recommended using closed-circuit television (CCTV) security cameras for social distancing monitoring and infection risk assessment (COVID-19). The suggested model has many components, including CSPDarknet53, neck, and head. The CSPDarknet53 consists of two components: CNN (you only look once, version 4) and the pre-trained Darknet53 model. The YOLOv3 model is present in the spatial pyramid pooling (SPP), path aggregation network (PAN), spatial attention module (SAM), and head block components. For training purposes, Microsoft Common Objects in Context (MS COCO) and Google Open Picture datasets were utilized. For assessment reasons, the Oxford Town Centre dataset was utilized. The model achieved 99.8 percent accuracy. Li et al. [75] created the COVID-19 categorization and detection method using chest X-ray images. The dataset was obtained via Kaggle. There are other classifications within the dataset, including negative for pneumonia, indeterminate, typical, and unusual appearance. You only look once, version 5 (Yolov5), was utilized for target location detection. The authors evaluated the precision using Faster RCNN and EfficientDet as comparisons. The suggested Yolov5 model exceeded expectations.

Yoshitsugu et al. [76] identified the symptoms and localized the afflicted region of COVID-19. The dataset comprises 6334 chest X-ray scans divided into four classes: pneumonia-free, usual appearance, uncertain appearance, and atypical appearance. Utilizing an EfficientNetB7 backbone for image classification applications, YOLOv5 was used to detect and localize symptoms.

**Table 2:** The overview of applied object detection methods for COVID-19.

Ref.	Problem Definition	Architecture	Sample	Performance
[77]	COVID-19 detection through the chest X-ray images	DarkNet-53 + YOLO-v3	4 classes (194 COVID, 1772 bacterial pneumonia, 583 normal, 493 viral pneumonia cases) 2 classes (2848 non-COVID, 194 positives, samples) Dataset augmentation applied	Accuracy (97.11 ± 2.71%) multi-class, 99.81% binary class
[78]	Social distancing monitoring system using mass video surveillance	Faster R-CNN, YOLO, SSD	PASCAL-VOC, MS-COCO, vision-based social media event analysis	mAP 86.8%, 84.7%, 44.5%
[79]	Personal protective equipment detection	YOLOv4	5327 images (face mask and shield, no face mask, hand gloves)	Precision 78%
[80]	Detecting COVID-19 related CT abnormalities	RetinaNet	DeepLesion, 32,120 axial CT slices (liver, lung, bone, abdomen, mediastinum, kidney, pelvis, and soft tissue)	mAP 91.28% (internal testing), 87.83% (External-Set-1), 71.48% (External-Set-2), 83.04% (External-Set-3)
[81]	Social distancing detector through thermal images or video streams	YOLOv2, Fast R-CNN, R-CNN	1575 (Various scenarios while walking, different body positions, running, sneaking, and and different motion speeds)	Accuracy 95.6%, 91.2%, 88.5%, (Dataset II 94.5%, 90.5%, 86.5%)
[82]	Detection of masks and human eye areas. Measurement of body temperature through thermal cameras	YOLOv5, Resnet-50	Dataset Celeba, Coco, Helen, IMM, Wider, Group Images, IIITD and beyond visible spectrum disguise, UL-FMTV, Terravic Facial Infrared, IRIS	Precision 96.65%, 78.7%
[83]	The indoor distance measurement method through the closed-circuit television	DeepSORT, YOLOv3, YOLOv4	MS COCO dataset	Accuracy (4FPS10 62.5%, 4FPS24 93.7 4FPS35 78.9% 4FPS50 83.3%) mAP 30.4%, 42.1%
[84]	Data labeling and annotation framework	mask R-CNN	750 CT images (COVID-19 positive, COVID-19 negative)	Accuracy (train, validation, and test 99%, 93.1%, and 0.8%)

The performance of the classification model exceeds that of the object detection model. COVID-19 lesion detection was created by Nurmaini et al. [85] utilizing CT scans. The dataset contains 419 CT scan images of SARS-CoV-2 infected individuals and 200 CT scan images of healthy individuals. The architecture is comprised of CNNs and Faster RCNNs. The CNN model consists of thirteen convolution layers and four maximum polling layers. The suggested model attained a mean average accuracy of 90.41 percent (mAP). COVID-19 classification and localization methodology was developed by Rajaraman et al. [86]. The authors used six different datasets, including Pediatric CXR, RSNA CXR, CheXpert CXR, NIH CXR-14, Twitter-COVID-19 CXR, and Montreal-COVID-19 CXR. The model has various steps: segmentation block, repeated specific transfer learning models, and class-selective relevance mapping (CRM)-based region of interest (ROI) localization. The custom U-net architecture was designed for segmentation purposes. VGG-16, VGG-19, Inception-V3, Xception, DenseNet-121, NasNet-Mobile, MobileNet-V2, and ResNet-18 were applied for knowledge transfer purposes. The CRM-based ROI localization have been applied to interpret predictions of individual convolutional neural networks and compare against the ground truth.

Using chest X-ray images, Saiz et al. [87] created the COVID-19 position detection algorithm. They collected data from Kaggle and GitHub. This set possesses two classes: one standard and the other COVID-19. They used a single-shot detector architecture to detect the position of an item. VGG-16 is the foundation of the single-shot multi-box detector network (SSD300). The model achieved a sensitivity of 94.92 percent and a specificity of 92 percent.

Loey et al. [88] suggested a method for detecting medical face masks. The data collection included 1535 images. To enrich the dataset, the data augmentation approach was utilized. ResNet-50 architecture is used to extract features, and the YOLO v2 method is used to recognize medical face masks. The model’s average degree of accuracy was 81%.

Li et al. [89] revealed severe instances and minor symptoms based on the identification of COVID-19 using CT scans of the lungs. The dataset was acquired from Zhongnan Hospital Wuhan University and the Italian Society of Medical and Interventional Radiology. The model is composed of the network-in-network technique with instance normalization. ResNet was used in COVID-19 CT images to extract features. The performed a performance comparison of domain-adaptive Faster R-CNN, Faster R-CNN, and few-shot adaptive faster R-CNN architectures. Their proposed model was superior.

In object detection, YOLO, SSD, and R-CNN were the major applied models. Figure 5 shows YOLO base models contributed 50%, then R-CNN 28.9%, SSD and others models contributed 10.5%. Table 2 presents a systematic overview of object detection methods for COVID-19.

### 4.2. Transfer Learning

Transfer learning applies a previously-trained model to a new problem, which is more accurate, quicker, and requires less training data. The transfer learning-based COVID-19 study includes automatic analysis of medical pictures, COVID-19 classification, identification of lung disease severity, and the automated COVID-19 screening model.

De Moura et al. [90] established automated classification methods for chest X-ray images. There are three groups in the dataset: COVID-19, pneumonia, and healthy patients. To enhance the visual data, the scaling with horizontal flipping augmentation approach was used. The authors employed six deep learning architectures for classification, including DenseNet-121, DenseNet-161, ResNet-18, ResNet-34, VGG-16, and VGG-19. When detecting COVID-19 pneumonia lessons, their method earned a 97.44 percent accuracy rate.

Abbas et al. [91] introduced a transfer learning-based CNN-based self-supervised sample decomposition method. Three datasets from various sources were utilized. The steps of this approach include sample decomposition, pretext training, and downstream task. Knowledge-transfer-pre-trained backbones such as ResNet18, GoogleNet, and VGG19 were utilized by the authors. The suggested model with the ResNet18 backbone exceeded expectations. Rehman et al. [92] investigated an CT and X-ray image-based categorization technique. The dataset includes 200 COVID-19, 200 viral pneumonia, 200 bacterial pneumonia, and 200 X-ray and CT images of healthy individuals. This research applied nine deep transfer learning architectures, such as Aexnet, SqueezeNet, GoogLeNet, VGG16, MobileNetv2, ResNet18, ResNet50, ResNet101, and DenseNet201. The total accuracy of the model was 98.75 percent. Ahuja et al. [93] reported a technique for detecting COVID-19 utilizing CT scan images and transfer learning. Two classes comprised the dataset: positive and normal. Data enhancement was utilized to boost the quantity of photographs. To transmit knowledge, ResNet18, ResNet50, ResNet101, and SqueezeNet designs were utilized. ResNet18 was utilized to determine the localization of abnormalities. ResNet18 performed better overall.

Shamsi et al. [94] created an uncertainty-aware transfer learning framework for detecting COVID-19 utilizing X-ray and CT images. The dataset was compiled from several sources. Four deep learning architectures, including InceptionResNetV2, VGG16, ResNet50, and DenseNet121, were utilized to extract features. To identify COVID-19 instances, extracted features were analyzed by many machine learning algorithms, including k-nearest neighbors (KNN), SVM, gaussian process, neural network (NN), random forest, adaboost, and naive bayes. The ResNet50 architecture with SVM and NN beat the competition.

To categorize chest X-ray images, Bassi et al. [95] built dense convolutional networks with transfer learning. There are three classifications in the dataset: COVID-19, pneumonia, and normal. The model contains two deep learning backbones, DenseNet 201 and DenseNet 121. They utilized twice transfer learning with output neuron technique to boost performance. Jaiswal et al. [96] examined the COVID-19 identification approach based on transfer learning. The authors acquired 2492 CT scan images from Kaggle for the dataset: 1262 are positive; 1230 are negative. To transfer information, four deep learning models, including VGG-16, ResNet152V4, InceptionResnetV2, and DenseNet201, were utilized. DenseNet201 outperformed VGG-16, ResNet152V2, and InceptionResnetV2 structures.

**Table 3:** The overview of existing transfer learning applications for COVID-19.

Ref.	Problem Definition	Architecture	Sample	Performance
[97]	COVID-19 classification	DensetNet201, ResNet101, CCSHNet	Category (COVID-19, CAP, SPT, HC) total 1164 CCT images	F1 score 95.53%, 96.74%, 97.04%
[98]	The deep transfer learning technique has used to classify COVID-19 infected patients	(CNN+ ResNet-50)	413 COVID-19 (+), 439 normal or pneumonia	Accuracy 93.01%, sensitivity 91.45%
[99]	An automated COVID-19 screening model	CNN, VGG-16 ResNet-50	219 COVID-19 positive, 1345 pneumonia infection and 1341 no infection	Accuracy 89.16%, 96.01%, 93.29%
[100]	Hybrid deep transfer learning-based COVID-19 positive cases detection using chest CT X-ray images	AlexNet, BiLSTM	COVID-19 219, Viral Pneumonia 1345, Normal 1341	Accuracy 98.14%, 98.70%
[101]	Transfer knowledge-based chest X-ray images classification. Random oversampling was applied to overcome the class imbalance problem	ResNet, Inception v2, (Inception + ResNet-v2), DenseNet169, NASNetLarge	COVID-19 108, other pneumonia 515, normal 533, tuberculosis 58	F1 sore 56%, 74%, 96%, 95%, 98%
[102]	GAN with deep transfer learning technique for coronavirus detection in chest X-ray images	Alexnet, Googlenet, Restnet18	Total 307 X-ray images (COVID-19, normal, pneumonia bacterial, and pneumonia virus)	Binary classes accuracy (99.6%, 99.9%, 99.8%)
[103]	Two-step transfer learning for COVID-19 detection	ResNet34	COVID-19 189, pneumonia 252, Normal 235 images	Accuracy 91.08%
[104]	Deep transfer learning-based COVID-19 detection using X-ray images	DenseNet201, Resnet50V2 and Inceptionv3	COVID (+) 538, COVID (−) 468	Accuracy 91.11%, 91.11%, 90.43%
[105]	COVID-19 screening in chest X-rays images	EfficientNet B0, EfficientNet B1, EfficientNet B2, EfficientNet B3, EfficientNet B4, EfficientNet B5, MobileNet, MobileNet V2, RESNET 50, VGG-16, VGG-19	13,800 X-ray images, Healthy, non-COVID-19 pneumonia, COVID-19 patients	Accuracy 90.0%, 91.8%, 90.0%, 93.9%, 93.0%, 92.2%, 90.4%, 90.0%, 83.5%, 77.0%, 75.3%
[106]	Multiple Kernels-Extreme Learning Machine-based DNN system to detect COVID-19 disease from CT scan images	AlexNet, GoogleNet, VGG16, MobileNetv2, ResNet18, Inceptionv3 (DenseNet201+ MK-ELM)	349 images of COVID-19 and 397 images of no-findings (data augmentation was applied to expand the dataset)	Accuracy 90.34%, 92.86%, 92.65%, 93.19%, 92.22%, 92.54%, 98.36%

Horry et al. [107] developed transfer learning for COVID-19 identification through X-ray, ultrasound, and CT scan images. To eliminate sampling bias, a normalization function and the contrast-limited adaptive histogram equalization (N-CLAHE) approach were utilized. To enrich the data, techniques such as horizontal flip, horizontal and vertical shift, and rotation were utilized. For training models, the VGG16, VGG19, ResNet50 V2, Inception V3, Xception, InceptionResNet V2, NasNetLarge, and DenseNet 121 deep learning backbones were deployed. The VGG19 model attained the highest precision of 86% for X-rays, 84% for CT scans, and 100% for ultrasound. Zhu et al. [108] investigated convolutional neural network (CNN) and knowledge-based transfer (VGG16) to predict lung disease severity scores. One-hundred and thirty-one chest X-ray images from 84 COVID-19-positive individuals make up the dataset. Conv2D, BatchNormalize, MaxPool2D, Flatten, Dropout, and Dense are the 15 CNN layers. The authors compared CNN and VGG16 models’ performances. The VGG16 architecture was superior.

Using chest radiographic images, Gianchandani et al. [109] developed a deep transfer learning-based COVID-19, viral pneumonia, and bacterial pneumonia classification technique. For experimental purposes, two datasets were utilized, one from Kaggle and the other from the University of Dhaka and Qatar University. For classification objectives, VGG16, ResNet152V2, InceptionresNetV2, DenseNet201, and the suggested ensemble (VGG16+DenseNet) were employed as deep learning backbones. Multiple dense, rectified linear unit (ReLU) activation, and dropout layers were added to the aforementioned DL models. The accuracy of VGG16+DenseNet was 96.15 percent.

Hira et al. [110] reported a technique based on deep learning to distinguish COVID-19 illness patients from bacterial pneumonia, viral pneumonia, and healthy cases. To enhance the image in the pre-processing phase, random horizontal flipping and random cropping were implemented. For binary and multiclass classification, the pre-trained models Inception ResNetV2, ResNeXt-50, GoogleNet, ResNet-50, Se-ResNet-50, AlexNet, Inception V4, DenseNet121, and Se-ResNet-50 were utilized. Among all the pre-trained models, the Se-ResNeXt-50 architecture had the highest accuracy for binary class and multi-class classification.

Ibrahim et al. [111] investigated the classification approach for chest X-ray images using the AlexNet architecture. The dataset includes 11568 images divided into four categories, including COVID-19, bacterial pneumonia, non-COVID-19 viral pneumonia, and normal CXR. Binary to multiclass classification was applied for classification purposes. The classification method with the highest accuracy was binary. Using chest X-ray images, Kamal et al. [112] constructed eight transfer knowledge architectures for COVID-19 classification. The dataset consists of 760 images classified into four categories: COVID-19, pneumonia, healthy, bacterial pneumonia, and viral pneumonia. For detecting purposes, eight pre-trained models were employed, including ResNet50, ResNet50V2, VGG19, InceptionV3, MobileNet, MobileNetV2, DenseNet121, and NasNetMobile. DenseNet121 outperformed the others.

Deep transfer learning classification techniques using meta-heuristic algorithms were created by Canayaz et al. [113]. The dataset includes three image classes: COVID-19, normal, and pneumonia. X-ray image characteristics were extracted utilizing AlexNet, VGG19, GoogleNet, and ResNet. Effective features were chosen using binary particle swarm optimization (BPSO) and binary grey wolf optimization (BGWO) metaheuristic methods. Selected characteristics were classified using the SVM method. The performance of the suggested model using the VGG19 architecture was superior. Das et al. [114] devised an algorithm for the automated analysis of chest X-ray images. CNN and deep transfer learning blocks are included in the suggested approach. CNN has initially extracted characteristics from chest X-ray scans. They utilized the Xception architecture to communicate knowledge. The suggested model was compared against SVM, random forest, backpropagation network, adaptive neuro-fuzzy inference system, CNN, VGGNet, ResNet50, Alexnet, Googlenet, and Inceptionnet V3. The proposed model gained improved precision.

The COVID-19 detection method was created by Rodriguez et al. [115] using spectrograms of coughing, sneezing, and other respiratory sounds. The dataset contains two labels, sick and not sick, gathered by the pharmaceutical manufacturer Pfizer in the United States. To train the model, an Xception pre-trained architecture was utilized. As a result of the overfitting problem, the model achieved poor performance.

Loey et al. [116] created a categorization algorithm of COVID-19 cough sound symptoms using scalogram images. This methodology ran its tests using the COUGHVID dataset. There are 755 COVID-19 and 702 healthy wave cough sounds in the collection. Data categorization was performed with GoogleNet, ResNet18, ResNet50, ResNet101, MobileNetv2, and NasNetmobile architectures. The ResNet18 design achieved high precision. Imran et al. [117] investigated the COVID-19 screening application by analyzing cough sounds. The authors utilized the ESC-50 sound dataset, which included 96 bronchitis coughs, 130 pertussis coughs, 70 COVID-19 coughs, and 247 normal coughs. Mel frequency cepstral coefficients (MFCC) and principal component analysis were used to extract features (PCA). The sound data was transformed into a mel-spectrogram, which represents the frequency spectrum. The presented model was divided into three parts: a deep transfer learning-based multi-class classifier, a classical machine learning-based multi-class classifier, and a deep transfer learning-based binary class classifier. This article does not specify which transfer learning model was used. The random undersampling approach was utilized to rectify the imbalance; however, information loss is a worry. Support vector machine classification model for structural data.

For transfer learning, Figure 6 illustrates ResNet based models achieved highest proportion in COVID-19 context. Nevertheless, VGG, DenseNet, and inception based models contributed 15.3%, 13.7%, and 9.9% respectively. Table 3 presents summary of transfer learning applications for COVID-19.

### 4.3. Image Segmentation

The segmentation approach provides the grouping of similar regions or segment maps as outputs corresponding to the input. Medical images based on COVID-19 infected area segmentation, lung and infection region segmentation, and infected tissue segmentation are key applications in this sector.

Saeedizadeh et al. [118] investigated the segmentation framework for COVID-19 chest area detection using CT images. The dataset was compiled from several sources. The authors created a TV U-Net architecture that resembles a U-Net design. Nine hundred images were utilized for the aim of evaluating models. The TV U-Net model earned a mIoU rate of 99 percent and a dice score of around 86 percent. Ma et al. [119] in the Corona Cases Initiative and Radiopaedia compiled a dataset with 300+ illnesses and 1800+ slices. Task one, three-class segmentation, including lung, infection, or both, was based on restricted annotations. Task two, two-class segmentation consisting of lung and infection, was based on non-COVID-19-CT-scan images. The third task, two-class segmentation—healthy lung and infected lung—was based on COVID-19 and non-COVID-19-CT-scans. Average dice similarity coefficient (DSC) scores for their model were 97.3%, 97.7%, and 67.33%.

Yazdekhasty et al. [120] demonstrated COVID-19-infected lung segment areas. The dataset was compiled from two sources open to the general public. The author has created a U-Net-based, two-dimensional model for two distinct segmentation kinds. One decoder was for the healthy lung area and another for the diseased lung region. The design comprised four building blocks: a decoder, two parallel encoders with a split structure, and a merging encoder. The model’s sensitivity was 74.9 percent, and its specificity was 99.7 percent. Ranjbarzadeh et al. [121] investigated CNN-based COVID-19 CT images that autonomously partition diseased lung tissues. The dataset was compiled from two sources open to the general public. They implemented fuzzy c-means clustering and local directed pattern encoder algorithms for performance enhancement. The model scored 96 percent accuracy, 97 percent recall, and 97 percent F-score.

Zheng et al. [122] designed the three-dimensional (3D) CU-Net architecture to detect COVID-19-infected regions in 3D chest-CT-scan images. The model of 3D CU-Net was built on a 3D U-Net architecture. Rich features were extracted with the aim of performance enhancement. Three variations of the model exist: 3D U-Net, 3D CU-Net (α=0.5,β=0.5), and 3D CU-Net (α=0.3,β=0.7). Chen et al. [123] investigated 3D U-Net architecture for COVID-19 segmentation using CT images. This work was evaluated using both public and private datasets. The private dataset contained 89 COVID-19 infection records. The public dataset had 1700 data points. Combination loss and data augmentation strategies were introduced to enhance the training impact. In comparison to other methods, the model yielded amazing results. Yan et al. [124] reported a 3D-based CNN segmentation approach for COVID-19-infected chest CT images. The collection included 165,667 annotated chest CT images from 861 COVID-19-positive individuals. The authors created the FV block that modifies the global parameters of the features for adaptively segmenting COVID-19 infection. The COVID-19 segmentation model received a dice score of 72.6%, whereas lung segmentation had a die score of 98.7%.

Frid-Adar et al. [125] examined the identification, segmentation, and grading of COVID-19 pneumonia in chest X-ray images. The authors employed a two-stage training approach for detection and segmentation. The ResNet50 model was employed for detection purposes. For segmentation purposes, a modified U-Net with pre-trained VGG-16 architectures was employed. Degerli et al. [126] suggested X-ray images based on the production and detection of the COVID-19 infection map. They are architectures that integrate segmentation and detection. For segmentation purposes, the U-Net, UNet++, and deep layer aggregation (DLA) algorithms were utilized. For detection purposes, the CheXNet, DenseNet-121, Inception-v3, and ResNet-50 algorithms were used. The model attained a sensitivity of 94.96 percent and a specificity of 99.88 percent.

**Table 4:** Review of image segmentation applied applications for COVID-19.

Ref.	Problem Definition	Architecture	Sample	Performance
[127]	CT image segmentation and classification	Dual path Network (DPN)-92, Inception-v3, ResNet-50, and Attention ResNet-50 FCN-8s, V-Net, U-Net, 3D U-Net++	Segmentation (positive 877, negative 541) Classification (positive 718, negative 70, and other diseases 343)	Sensitivity 97.4%, Specificity 92.2%
[128]	Automatic segmentation of lung opacification from CT images	SCOAT-Net, PSPNet, ESPNetv2, DenseASPP, UNet+, DeepLabV3+, U-Net, COPLE-Net, CE-Net, Attention U-Net	Two patients scanned at different times, and Kaggle dataset	Proposed model (DSC 88.99%, Sensitivity 87.85%, PPV 90.28%)
[129]	COVID-19 lesion segmentation in CT slices	Dilated dual attention U-Net architecture with a ResNeXt-50	Three open-source datasets total 1645 slices	Dice 72.98%, recall 70.71%
[130]	Segment the radiological images	Superpixel based fuzzy modified flower pollination	115 CT scan images	—
[131]	ML and DL-based classifier with CT image opacity map	3D neural network, DenseUnet	2446 chest CTs images	AUC 93%, sensitivity 90%, specificity 83%
[132]	Multi-point supervision network for segmentation of COVID-19 lung infection using CT image	U-Net based (MPS-Net)	300 CT images	Dice 83.25%, sensitivity 84.06%, specificity 99.88%, IOU 74.2%
[133]	Binary and multi-class detection and labeling of infected tissues on CT lung images	SegNet and U-NET	100 CT images	Binary segmentation (SegNet) 95%, multi-class (U-NET) 91% mean accuracy
[134]	Lung and lobar segmentation of CT images in patients with COVID-19	Seg3DNet	A combination of human and animal 3D CT images. 1453 for training, 7998 for evaluation	Dice coefcient of 0.985 ± 0.011
[135]	The segmentation and classification of COVID-19 using chest X-ray (CXR) images	U-Net	1645 CXR images	F1-Score (binary 88%, multiclass 83%)
[136]	COVID-19 classification using plain and segmented lung CXRs	U-Net, Modified U-Net	COVID-19 3616, Normal 8851, Non-COVID 6012	Dice 96.3%, 96.94%

Fung et al. [137] described a two-stage deep learning algorithm for segmenting COVID-19 chest CT images. They implemented three models for segmentation: U-net, single SInfNet, and SSInfNet. The SSInfNet model consists of one U-net and one SInfNet. The single SInfNet model fared better. Wu et al. [138] presented a combined classification and segmentation method for the COVID-19 chest CT diagnosis. They produced a big dataset consisting of 144,167 chest CT scans of 400 COVID-19 patients and 350 uninfected cases. There were 3855 CT scans from 200 COVID-19 patients. The Res2Net classification model was utilized for classification purposes. In the encoder block, the VGG-16 model was utilized. Encoder and decoder blocks utilized a U-shaped design. The model achieved 95.0% classification sensitivity and 93.0% classification specificity. The score on the segmentation dice was 78.5 percent.

Laradji et al. [139] presented a consistency-based loss function for the segmentation of COVID-19 in CT images. The model consists of two branches, the first of which encodes, and the second decodes the altered input. The authors employed three open-source medical segmentation datasets for assessment purposes. CB (Flip, Rot) + PL identified the suggested model with superior performance.

Wang et al. [140] suggested a method for learning using a hybrid encoder. The authors gathered data from four distinct sources, including the Corona Cases Initiative and Radio Media, Medical Segmentation Decathlon (MSD) Lung Tumor, Structseg Lung Cancer, and Non-Small Cell Lung Cancer (NSCLC) databases. For encoder–decoder blocks, the 3D U-Net architecture was utilized. The authors utilized transfer learning techniques, such as continuous learning, body fine-tuning, and pre-trained lesion representations. The model attained DSC values of 0.704, normalized surface distance (NSD) values of 0.735, sensitivity values of 0.682, F1-score values of 0.707, accuracy values of 0.994, and MCC values of 0.716.

Using a chest CT image, Oulefki et al. [141] created an algorithm for the segmentation and quantification of COVID-19 lung infection. This approach combines linear and logarithmic stitching with multilayer, parametric thresholding. The author proposed a superior method to U-Net, Attention-UNet, Gated-UNet, Dense-UNet, U-Net++, Inf-Net GraphCut, Watershed, and medical image segmentation (MIS) designs.

For COVID-19 diagnosis, Gao et al. [142] developed dual-branch combination network (DCN) classification and lesion segmentation algorithms. For validation purposes, internal and external datasets were utilized. The external dataset includes CT images from 1795 people. The concept is comprised of numerous components, including U-net, ResNet-50, and fully linked three-layer networks. ResNet-34, ResNet50, ResNet101, VGG-16, and DenseNet-121 were the conventional deep learning models used in the performance comparison. Their U-net and ResNet-50 hybrid model surpassed the competition.

U-Net-based models were the major driven method for image segmentation. Figure 7 reveals U-Net based model contributed 55.4% in this domain. However, DeepLabv3/DeepLabv3+, CNN, FCN-8, SegNet, supervised InfNet and other models contributed rest of the proportion. Table 4 presents overview of image segmentation applied methods for COVID-19.

### 4.4. Few/One-Shot Learning

Unlike humans, machine learning and deep learning models require a huge number of instances in order to tackle novel problems. The shot learning technique can learn items from one or a small number of samples. Detection of COVID-19 and medical image analysis are the primary applications in this arena.

Aradhya et al. [143] developed a cluster-based one-shot learning technique for recognizing COVID-19 in chest X-ray images. A generalized regression neural network (GRNN) and probabilistic neural network (PNN) were used to form the model (PNN). For their investigation, the authors utilized 306 images divided into four classes. For model assessment, they offered five clusters based on class and image sample variation. The model’s detection accuracy ranged from 61.84 percent to one hundred percent.

Using Siamese networks, Jadon et al. [144] investigated a few-shot learning approach for detecting COVID-19. They utilized two datasets, one from various Asian universities and the other from the University of Montreal. The Siamese networks have identical characteristics and weight distributions. The Siamese networks attained a detection accuracy of 94.6 percent.

Jiang et al. [145] reported a COVID-19 CT diagnosis approach based on supervised domain adaptation. The authors’ network architecture was Siamese. The suggested method consists of three branches: the source branch, the target branch, and the prediction branch. They used five cases with shot numbers 1, 3, 5, 7, and 9 for assessment reasons. The model’s accuracy was 80.40 percent, and its F1 score was 79.98 percent.

The few-shot-learning-based double-view matching network that identifies COVID-19 in X-ray images was built by Szűcs et al. [146]. The network for matching is comprised of metric learning and the lazy learner. The authors utilized 680 data from 15 classes for experimentation purposes. Their custom model outperformed both the matching network and the KNN.

Aradhya et al. [147] suggested one-shot learning for chest X-ray image categorization. One-shot learning is comprised of probabilistic neural networks and entropy used to describe the input image’s texture. Note that 300 and 24,314 images were examined for training and testing, respectively. The model scored 96.4 percent precision, 96.6 percent recall, 96.0 percent F-measure, and 96.4 percent accuracy.

Chen et al. [148] investigated the categorization of COVID-19 utilizing chest CT scans based on a few training sample shots. For random cropping and random cropping with color distortions, the authors utilized a stochastic data augmentation approach. The model includes the encoder, projection head, and contrastive loss function, among other components. They compared its performance to those of conventional deep learning models, such as ResNet-50 and DenseNet-121. The suggested model was 86.8 percent accurate.

Shorfuzzaman et al. [149] introduced automated COVID-19 case detection. The model is composed of metric-based methods, such as the Siamese network. Nearest neighbor approaches and kernel density estimates make up the components of metric-based methods. Utilizing a convolutional neural network, the characteristics of two images were extracted, and their similarity determined. Thirty and six-hundred and forty-eight CXR images were considered for training and testing, respectively. The performance of the suggested Siamese network model is superior to that of existing CNN models, including InceptionV3, Xception, Inception, ResNetV2, and VGG-16.

**Table 5:** Meta-analysis.

Objective	Reference	Number of Studies	Main Reason for Implementation	Technical Issue Faced
**Medical Image Analysis**	[32,43,47,55,57,59,60,62,63,65,72,73,75,76,77,80,84,85,86,87,89,90,92,93,94,95,96,98,99,100,101,102,103,104,105,106,107,108,109,110,111,112,113,114,118,119,120,121,122,123,124,125,126,127,128,129,130,131,132,133,134,135,136,137,138,139,140,141,143,144,145,146,147,148,149,150]	76	COVID-19 classification and detection. Point-of-care COVID-19 diagnostic. Infected tissues identification. Infection measurement. Abnormalities detection. MERS, influenza, pneumonia classification. Lung disease identification. Infected or not infected classification. Infection map generation. Intelligent healthcare systems. Localization of the affected area. Control spread of COVID-19 infection.	Overfitting underfitting. Hyper-parameter tuning issues. Physical resource limitation. Imbalanced dataset. Synthetically generated images.
**Clinical and socio** **demographic data analysis**	[20,22,23,24,25,27,28,29,30,31,35,36,40,42,44,46,54]	17	COVID-19 detection. COVID-19 case forecasting. COVID-19 infection prediction. Patients mortality risk prediction. Mechanical ventilation management. Risk factor analysis. Patient intensive care prediction. Early risk identification. Clinical prognosis evaluation. Patient severity and intubation prediction,	Data inconsistencies and noise. Insufficient medical information. Lack of external data. Imbalanced dataset. Bias in distribution.
**Sound analysis**	[37,38,51,115,116,117]	6	COVID-19 detection. Cough classification. Symptoms classification. Bronchitis, pertussis identification.	Overfitting. Low performance. Lack of evaluation. Synthetically generated. Limited physical resources. Imbalanced dataset.
**Genetic analysis**	[26,48,58,61]	4	COVID-19 disease-related genes detection. Identification of the critical cytokines. Extract associations for coronavirus infectious diseases.	Synthetically generated data. Imbalanced dataset.
**Protective equipment observation**	[66,67,68,69,70,79,82,88]	8	Face mask, face shield, glasses, gloves detection. Facial recognition. Measurement of body temperature.	Lack of proper evaluation and performance. Synthetically generated images. Overfitting and underfitting.
**Social control**	[71,74,78,81,83]	5	Detection of social distancing violations. Infected person detection. Maintaining a safe distance. Public place monitoring. Live object count. Infection risk assessment. Reduction of the spread of the coronavirus.	Synthetically generated images. Low speed for data processing.

**Table 6:** Current challenges and future research directions.

No.	Challenges	Current Limitation	Future Research Directions
1	Physical resource and computing time	Most deep learning models require more data and training time.	Developing metric learning, meta-learning, plug-and-play modules, optimization, and probability-based methods to overcome training time and physical resources challenges.
2	Bias	Many models are trained or tested by the unrepresentative reality or biased data.	Applying bias mitigation methods including optimized preprocessing, fair data adaptation, meta-algorithm for fair classification, adversarial debiasing, rich subgroup fairness, exponentiated gradient reduction, grid search reduction, etc.
3	Embedded machine learning	The embedded machine learning approach has still absent.	Design sophisticated machine learning approach combination of low latency, reduced power consumption, improved environmental performance, network bandwidth efficiency, and strong privacy.
4	Drugs and vaccine development	Requires to identify the most relevant biotargets and large-scale training datasets.	Focusing on protein-coding, mRNA sequence design, molecule generation, developing general vaccine prototypes, and predicting the response of the immune system.
5	Limited uses of ultrasound data	A few studies used ultrasound images.	Implementing segmentation and shot learn methods through the ultrasound image for the specific task.

Karnes et al. [150] created a point-of-care COVID-19 ultrasound imaging diagnostic system. Few-shot learning was applied to create encoded disease state models. A deep neural network (DNN) was utilized for feature extraction. Calculated Mahalanobis distances were used for image categorization.

In shot learning, Figure 8 shows siamese network contributed 37.5%, matching network 12.5%, probability based model and others contributed 25%. Table 5 presents the meta-analysis for specific domains and Table 6 provides current challenges, limitations as well as future research directions.

## 5. Discussion

The aforementioned sections where we systematically reviewed papers and their contribution in the field of AI with respect to COVID unequivocally provide us with futuristic insights related to upcoming endemic and pandemics. It is evident that AI has been widely used to tackle the COVID pandemic, and the insights generated from this incident possesses wider implications for forthcoming disasters in health domain. Before we discuss the policy implications for future pandemics or endemics in general, it is important to understand the past and the present.

According to research conducted by [2], a number of pandemics and epidemics have emerged around the globe since 541, when the Justinian disease became the first pandemic on Earth. With a mortality toll of 100 million in the then-powerful Roman Empire, the epidemic contributed significantly to the final weakening and consequent collapse of the Roman and Byzantine empires, as shown by [151]. Until the late 19th century, the second pandemic, often known as the black death, killed 200 million people and is still regarded as one of the deadliest plagues in recorded history [152]. According to [2] the Black Death (1347–1351) wiped out at least 30 percent of Europe’s population and was followed by waves such as the plague of Milan (1630), the great epidemic of London (1665–1665), and the plague of Marseille (1720–1722). Faruque et al. [153] also highlight that the pre-digital and post-industrial revolution eras have seen a number of influenza pandemics, among which the cholera pandemic in the 19th century and the Spanish flu (1918–1919) were notable for their nature, scope, and variability, and contributed significantly to the number of deaths in the industrial revolution period. Over the course of nine months, three different waves of the 1918–1919 influenza pandemic were seen to spread. In spring and summer in 1918, the first wave produced significant morbidity and low fatality. Nevertheless, the second and third waves had high death rates. The Spanish flu infected around 500 million individuals globally and was linked to 50 million deaths [154].

The reason the history is important is that we can understand how some deaths could be curbed if history lessons and insights from the past are efficiently implemented in the future.

One of the future implications of research is general-flu type disease detection. The flu season usually occurs in the fall and winter. It can be modeled for flu forecasting and flu detection methodologies by utilizing machine learning, deep learning, and AI in general. AI can also be utilized for modeling cholera, diarrhea, and several water-borne diseases as well. Our implication shows that, since AI works on the basis of data which may be audio, video, text, tabular, or image-based (etc.), all of these data which are specific to a disease can be modeled for forecasting, prediction, and generating policy implications and guidelines in the public health domain. The source of water may be modeled to understand a good or bad source of water to limit the water-borne endemics. Similarly, in Asian countries, particularly Bangladesh and Afghanistan, people are severely prone to mosquito-related diseases and deaths due to unsanitary conditions that they are exposed to. The images of mosquitoes can be processed to understand the genera of the mosquitoes, and there can be models for specific regions to realize their origins and breeding processes. The insights generated from this AI -ased model can be used by policymakers and stakeholders to limit the growth of such insects which eventually would curb the dengue, culex, chikungunya, or AIDS endemic that are prevailing in South Asian countries.

Monkeypox disease can also be considered endemic in general due to its severity. It came after COVID-19. AI can also play a substantial role in curbing this disease by modeling the symptoms, causes, and effects of the disease, which was previously done for COVID-19. Several time series forecasting methods, image processing, and video processing-based detection methods may be employed to limit the growth of monkeypox.

As we have seen in COVID-19-related research, AI was utilized in developing intelligent applications that provide information related to treatment, facilities, and procedural guidance for things such as quarantine, vaccine information, and so forth. There are AI applications that also detect whether a person is wearing a mask or not. Accumulating all these developments from the COVID-19 era where AI contributed directly can be mapped to future endemics of the COVID-19 genre, such as Middle Eastern Respiratory Syndrome (MERS) and Severe Acute Respiratory Syndrome (SARS), to direct policy guidelines and provide insights for future generations of stakeholders to lessen upcoming pandemics and endemics.

## 6. Conclusions

The COVID-19 pandemic’s vulnerability might trigger a serious worldwide calamity in the future. It has received great attention among certain scholars, corporate industries, and government organizations across the globe, since the epidemic has impacted a significant section of the world’s population. In this study, a comprehensive review of artificial intelligence models and various analysis has been demonstrated. Problem definition, reason for implementation, data source, models, and performance were examined for supervised, unsupervised, and few-shot learning. A detailed comparison of COVID-19 prediction models and various object detection methods, including SSD, YOLO, R-CNN, and other models, along with their accuracy and sensitivity performances, was shown. The existing transfer learning techniques for different architectures, including ResNet, DenseNet, VGG, Inception, AlexNet, and CNN, were the main applications for COVID-19 that have been studied and discussed in this work. Moreover, this work also showed various image segmentation techniques and models, and their sensitivity performances. However, this work also discussed the limitations of physical resources; the lack of proper evaluation; and the limitation of some technical issues, including data inconsistencies and noise, bias in distribution, synthetically generated data, and insufficient medical information. In the future, it is advised to carefully investigate artificial intelligence for COVID-19 prediction methodologies that have yet to be fully realized, and consider of their benefits and limitations. Several unique strategies against COVID-19 have attracted a significant deal of interest, even though they are still plagued by difficulties of complexity. To mitigate the complexity of the classical approaches against COVID-19, we should concentrate on the fairness of frameworks and large-scale datasets for training; and identifying the most relevant biotargets and sophisticated machine learning approaches that combine low latency, reduced power consumption, and strong privacy.

## Data Availability

Not applicable.

## Abbreviations

The following abbreviations are used in this manuscript:

AI	Artificial intelligence
DL	Deep learning
ML	Machine learning
KNN	K-nearest neighbors
SVR	Support vector regression
MLP	Multi-layer perceptron
PMLP	Polynomial multi-layer perceptron
PASC	Post-acute sequelae of COVID-19
ANN	Artificial neural networks
RT-PCR	Reverse transcription polymerase chain reaction
MAPE	Mean absolute percentage error
ARDS	Acute respiratory distress syndrome
DNN	Deep neural network
LSVC	Left superior vena cava
CT	Computerized tomography
SARS	Severe acute respiratory syndrome
MERS	Middle east respiratory syndrome
t-SNE	T-distributed stochastic neighbor embedding
PM	Particulate matter
DBSCAN	Density-based spatial clustering of applications with noise
SPGC	Signal processing grand challenge
VGG	Visual geometry group
RNN	Recurrent neural network
PCAUFE	Principal-component-analysis-based unsupervised feature extraction
RNA	Ribonucleic acid
SOFM	Self-organizing feature maps
U-Net	U-shaped convolutional neural network
GAN	Generative adversarial network
YOLO	You only look once
IAVP	Influenza-A viral pneumonia
X-ray	X-radiation
CAD	Computer-aided design
CCTV	Closed-circuit television
SAM	Spatial attention module
SPP	Spatial pyramid pooling
PAN	Path aggregation network
MS COCO	Microsoft Common Objects in Context
R-CNNs	Region-based convolutional neural networks
ROI	Region of interest
CRM	Class-selective relevance mapping
SSD	Single shot multibox detector
VOC	Visual object classes
mAP	Mean average precision
CelebA	CelebFaces attributes dataset
Wider	Web image dataset for event recognition
NN	Neural networks
N-CLAHE	Normalization function and the contrast limited adaptive histogram equalization
BPSO	Binary particle swarm optimization
BGWO	Binary gray wolf optimization
ESC	Environmental sound classification
DLA	Deep layer aggregation
3D	Three dimensions
NSD	Normalised surface distance
DSC	Dice similarity coefficient
PPV	Positive predictive value
IoU	Intersection over union
CXR	Chest X-ray
GRNN	Generalized regression neural network
PNN	Probabilistic neural network
